# A pancreatic tumor-specific biomarker characterized in humans and mice as an immunogenic onco-glycoprotein is efficient in dendritic cell vaccination

**DOI:** 10.18632/oncotarget.4359

**Published:** 2015-06-08

**Authors:** Aurélie Collignon, Adriana Teodora Perles-Barbacaru, Stéphane Robert, Françoise Silvy, Emmanuelle Martinez, Isabelle Crenon, Sébastien Germain, Stéphane Garcia, Angèle Viola, Dominique Lombardo, Eric Mas, Evelyne Béraud

**Affiliations:** ^1^ Aix-Marseille Université, CRO2, Centre de Recherche en Oncologie biologique et Oncopharmacologie, Marseille, France; ^2^ Inserm, UMR_S 911, Marseille, France; ^3^ Aix-Marseille Université, CNRS, CRMBM, Centre de Résonance Magnétique Biologique et Médicale, UMR 7339, Marseille, France; ^4^ Aix-Marseille Université, VRCM, Vascular Research Center of Marseilles, Marseille, France; ^5^ Inserm, UMR_S_1076, Marseille, France; ^6^ APHM, Hôpital Nord, Laboratoire d'Anatomie-Pathologie, Marseille, France; ^7^ Aix-Marseille Université, Marseille, France

**Keywords:** active immunotherapy, biomarker, cancer vaccines, pancreatic cancer, tumor-associated antigen

## Abstract

Oncofetal fucose-rich glycovariants of the pathological bile salt-dependent lipase (pBSDL) appear during human pancreatic oncogenesis and are detected by themonoclonal antibody J28 (mAbJ28). We aimed to identify murine counterparts onpancreatic ductal adenocarcinoma (PDAC) cells and tissue and investigate the potential of dendritic cells (DC) loaded with this unique pancreatic tumor antigen to promote immunotherapy in preclinical trials. Pathological BSDLs purified from pancreatic juices of patients with PDAC were cleaved to generate glycosylated C-terminal moieties (C-ter) containing mAbJ28-reactive glycoepitopes. Immunoreactivity of the murine PDAC line Panc02 and tumor tissue to mAbJ28 was detected by immunohistochemistry and flow cytometry. C-ter-J28^+^ immunization promoted Th1-dominated immune responses. *In vitro* C-ter-J28^+^-loaded DCskewed CD3^+^ T-cells toward Th1 polarization. C-ter-J28^+^-DC-vaccinations selectively enhanced cell immunoreactivity to Panc02, as demonstrated by CD4^+^- and CD8^+^-T-cell activation, increased percentages of CD4^+^- and CD8^+^-T-cells and NK1.1^+^ cells expressing granzyme B, and T-cell cytotoxicity. Prophylactic and therapeutic C-ter-J28^+^-DC-vaccinations reduced ectopic Panc02-tumor growth, provided long-lasting protection from Panc02-tumor development in 100% of micebut not from melanoma, and attenuated progression of orthotopic tumors as revealed by MRI. Thusmurine DC loaded with pancreatic tumor-specific glycoepitope C-ter-J28^+^ induce efficient anticancer adaptive immunity and represent a potential adjuvant therapy for patients afflicted with PDAC.

## INTRODUCTION

Pancreatic ductal adenocarcinoma (PDAC) remains one of the most universally lethal diseases, with a 5-year survival rate below 5% [1]. Pancreatic cancer is expected to become the second leading cause of cancer-related death by 2030 [2]. Combined chemotherapy provides a median survival of 11 months for patients with metastatic pancreatic cancer [3]. Although surgery and adjuvant chemotherapy have increased the 5-year survival rate to 15-20%, new treatments are needed. Among the promising approaches is cancer immunotherapy, particularly postsurgical vaccination with tumor antigen-loaded dendritic cells (DC) [4-6]. Cell transformation is often associated with modifications of the glycan moiety of membrane glycoproteins. Aberrant glycosylation of cell surface mucins therefore distinguishes neoplastic from normal cells. Such glycoproteins are challenging antigens since glycan alteration without change in the polypeptide backbone can create cell neo-antigens and affect their interactions with the immune system [7, 8].

We studied fucose-rich glycovariants of bile salt-dependent lipase (BSDL), which appear during fetal development and pancreatic oncogenesis processes [9, 10] and are characterized by monoclonal antibody J28 (mAbJ28) immunoreactivity (BSDL-J28^+^) [10, 11]. BSDL-J28^+^ is expressed in human pancreatic tumors [12] and cell lines [13] and so designated pathological (p)BSDL-J28^+^; non-tumor pancreatic tissue or cells do not express pBSDL-J28^+^ [9, 12]. The mAbJ28 recognizes carbohydrate-dependent antigenic structures, termed J28 glycotopes, located within the O-glycosylated mucin-like C-terminal moiety (C-ter-J28^+^) of pBSDL-J28^+^ [14]. Formation of this J28 glycotope, characterized by fucosylated O-linked side chains, requires core2 β1,6-N-acetylglucosaminyltransferase and α1,3/4-fucosyltransferase, two glycosyltransferases showing upregulated expression during pancreatic neoplastic processes [13].

Little information is available concerning pBSDL immunogenicity. We previously reported the presence of circulating antibodies that recognize the O-glycosylated-C-ter in most type 1 diabetic patients and in some patients afflicted with PDAC [15], which may reflect the potential of O-glycosylated C-ter-J28^+^ to induce humoral immunity. The nature of the tumor glycans may influence the uptake of the tumor-associated-carbohydrate antigens (TAAs) by immature DC, and thus affect the presentation to naive T cells of glycopeptides loaded onto MHC molecules, as well as DC maturation and function [16]. We showed that, unlike the TAAs MUC1 and HER2/Neu [17], which present structures that are differently glycosylated, pBSDL-J28^+^ can be delivered into the HLA class II pathway [12]. Thus, the expression of pBSDL-J28^+^ restricted to pancreatic tumor cells and tissues as well as their immunogenic potential led us testing the potential use of glycotope-J28^+^-loaded DC in DC-immunotherapy against PDAC. In this respect, we recently demonstrated that DC loaded with pBSDL-J28^+^ or C-ter-J28^+^ induce human T-lymphocyte activation [18].

Establishing the proof-of-concept of DC-vaccination in a murine model required the demonstration that (i) pBSDL-J28^+^ epitopes are common to humans and mice, (ii) pBSDL is immunogenic in mice, and (iii) C-ter-J28^+^-loaded DC trigger adaptive immunity against tumor cells. We succeeded in validating the use of C-ter-J28^+^ for DC-vaccination.

## RESULTS

### Immunoreactivity of murine pancreatic adenocarcinoma Panc02 cells and tumors to mAbJ28

To confirm that the monoclonal antibody (mAb)J28 specifically recognizes the glycoepitope located within the O-glycosylated C-ter of human pBSDL, we used transfected HEK-C-ter-6R cells. The polyclonal antibodies (pAbs) L64 and L32, reactive against physiological and pathological BSDLs (1A), and mAb J28, in a dose-dependent manner, detected C-ter-6R on non-permeabilized HEK-C-ter-6R cells but not on HEK-293T cells. We used these Abs to detect immunoreactivity on murine Panc02. All three Abs revealed a clear immunoreactivity (1B); intact or permeabilized Panc02 cells were stained with mAbJ28 indicating the presence of the epitope J28 in mouse as in human pancreatic tumor cells [19]. The expression of the J28 epitope on Panc02 was confirmed by immunocytochemistry, using HEK-C-ter-6R as positive control (1C) and HEK-293T and melanoma B16-F0 as negative controls. Next, slices of Panc02-tumor showed mAbJ28 immunoreactivity unlike those of normal pancreas under the same conditions (1D). As expected, tumors induced by HEK-C-ter-6R but not HEK-293T from nude mice displayed strong mAbJ28 staining. We also tested pancreatic tumors from genetically engineered mice (*Pdx1-Cre; Kras*^*G12D*^*; Ink4a/Arf*^flox/flox^ and *Pdx-1-Cre; LSL-Kras*^*GD12*^*; LSL-Trp53*^*R172H*^) developing PDAC, which showed no reactivity to mAbJ28 (unpublished data). Collectively these data demonstrate that mAbJ28 specifically reacts to epitope(s) localized at the O-glycosylated C-terminal domain of human and murine pBSDL expressed by both pancreatic cancer cells and inoculated cell-induced tumors.

**Figure 1:**
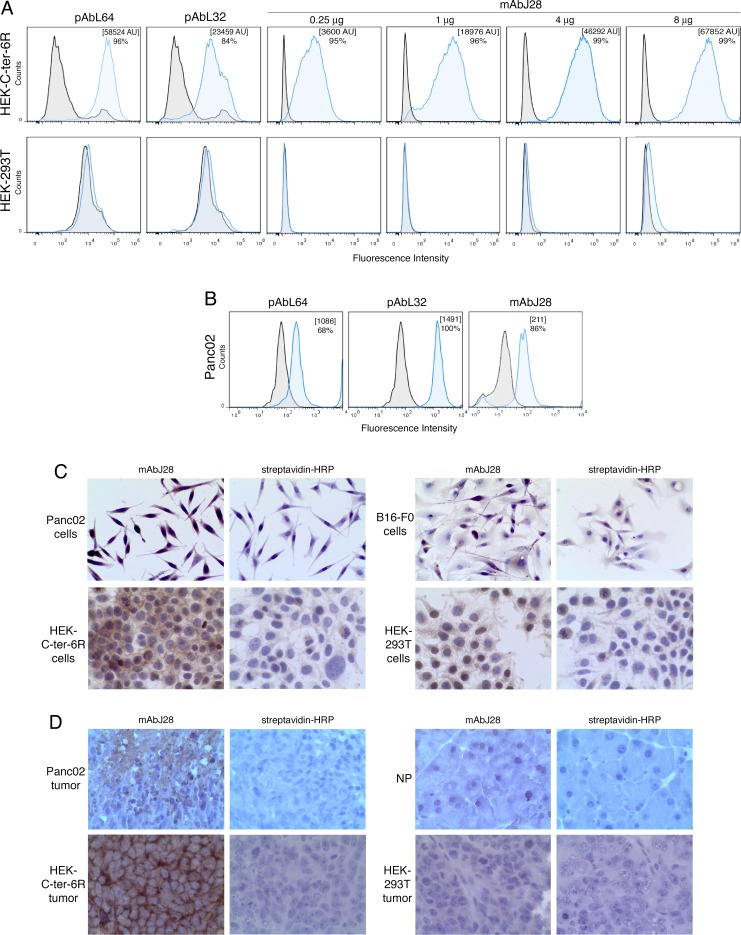
Immunoreactivity of C-ter-6R-transfected HEK cells and murine pancreatic adenocarcinoma Panc02 cells to mAbJ28 **A.** Expressions of C-ter-6R and J28 epitope on HEK-C-ter-6R and HEK-293T cells were analyzed by flow cytometry using pAbL32 (4μg), pAbL64 (4μg) and mAbJ28 (at different concentrations). **B.** Expression of pBSDL and J28 epitope on Panc02 cells was analyzed using pAbL64 (4μg), pAbL32 (4μg) and mAbJ28 (4μg). Black histograms represent non-specific binding using control isotype and blue histogram specific Ab as indicated. Representative images of immunohistocytochemical stainings with mAbJ28 **C.** on Panc02, HEK-C-ter-6R, B16-F0 and HEK-293T cells on polylysine-coated slides, and **D.** on 4% formalin-fixed, paraffin-embedded sections from tumors induced by Panc02, HEK-C-ter-6R and HEK-293T cells, and mouse normal pancreas (NP). Non-specific staining of streptavidin-HRP was used as internal control. Original magnification 250x. The results are representative of at least two independent experiments.

### Immunogenicity of glycoprotein pBSDL-J28^+^ and C-ter-J28^+^: CD4^+^ and CD8^+^ T-cells from mice immunized with full-length pBSDL-J28^+^ proliferated in the presence of either pBSDLs, from patients suffering PDAC, or the O-glycosylated C-terminal moiety

We investigated the ability of pBSDLs to promote adaptive immunity in mice. T-cells from mice immunized with pBSDL proliferated in the presence of either pBSDLs from different origins, or C-ter-J28^+^ (with optimal concentrations likely below 0.16 μM), with increased percentages of T-cells producing IFN-γ and granzyme B, and increased level of secreted IFN-γ (Supplementary Figure S1A-D). T-cells from mice immunized with C-ter-J28^+^ markedly proliferated in the presence of pBSDL and glycosylated C-ter-J28^+^; no such proliferation occurred in the presence of either a synthetic peptide encompassing an aa sequence identical to that of the C-ter or the short peptides EAT and GAP mimicking the most represented repeated sequences of BSDL (2A), thus underlining the requirement of glycosylated structures. C-ter-J28^+^ and pBSDL at the same molarity led to similar levels of proliferation of CD4^+^ and CD8^+^ T-cells (2B). Thus, T-cells from mice immunized with either one of the immunogens recognize both glycosylated antigens, but not non-glycosylated peptides. T-cell proliferation was associated to a time-dependent IFN-γ secretion; activation by C-ter-J28^+^ induced a greater level of secretion than that by pBSDL (2C). These data imply that immunodominant glycoepitope J28-bearing pBSDLs and glycosylated C-ter trigger CD4^+^ and CD8^+^ T-cell proliferation and IFN-γ secretion.

**Figure 2:**
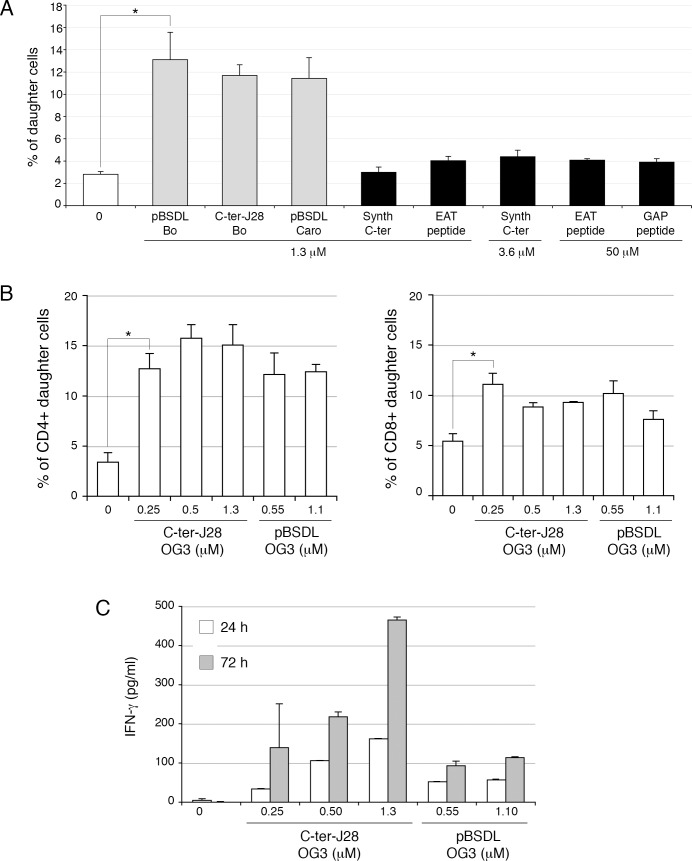
Glycosylated C-ter-J28^+^ induced activation of CD4^+^ and CD8^+^ T-cells in LN from mice immunized with C-ter-J28^+^ **A.** Isofluorane-anesthetized C57BL/6J mice (12 weeks old) were immunized at the base of the tail with 0.2 ml of emulsion containing 20μg of C-ter-J28^+^ of pBSDL-Caro in PBS and incomplete Freund's adjuvant supplemented with *Mycobacterium tuberculosis* (5mg/ml). They received 300 ng of *Pertussis* toxin (Pt) (Sigma-Aldrich) IP, before the immunization, and again 24 h later. CFSE-labeled cells from draining LN were cultured with different pBSDLs, C-ter-J28^+^ from Bo, synthetic C-ter or synthetic peptides EAT or GAP. After 6 days, CFSE dilution was analyzed by flow cytometry and T-cell proliferation evaluated. **B.** Cells were cultured at 2×10^5^ cells/well with C-ter-J28^+^ of pBSDL-OG3 or full-length pBSDL-OG3 at different molarities. After 6 days, cells were labeled with anti-CD4 and anti-CD8. CFSE dilution was analyzed by flow cytometry and T-cell proliferation evaluated. **C.** Culture supernatants were collected after 24 and 72 h for IFN-γ detection. The results are representative of four independent experiments. (*P < 0.05).

### DC pulsed with the O-glycosylated C-ter-J28^+^ triggered activation of CD3^+^ T-cells from mice immunized with C-ter-J28^+^ of pBSDLs

To delineate the ability of DC pulsed with C-ter-J28^+^ to activate T-cells, DC were loaded with C-ter-J28^+^ and underwent maturation with lipopolysaccharide (LPS) and CD40L. Mature (m)DC presented enhanced expression of the co-stimulatory and Class II molecules by comparison with DC cultured in control medium so-called immature (iDC) (3A). Concerning cytokine/chemokine profile, IL-12 (p40p70), RANTES, MCP-1 and IL-6 but no IL-2, IFN-γ, TNF-α, VEGF, or IL-10 were detected among the factors secreted by C-ter-J28^+^-pulsed mDC (3B). A positive spot for MCP-1 was found for iDC but less than that for C-ter-J28^+^-mDC. High amounts of IL-12 were secreted only by mDC, whether antigen-pulsed or not (3C), while IL-15 was secreted by mDC and iDC (3D). Remarkably, antigen-loading of DC impaired neither the increase in co-stimulatory and Class II molecules nor IL-12 and IL-15 secretion (3A, 3C and 3D).

**Figure 3:**
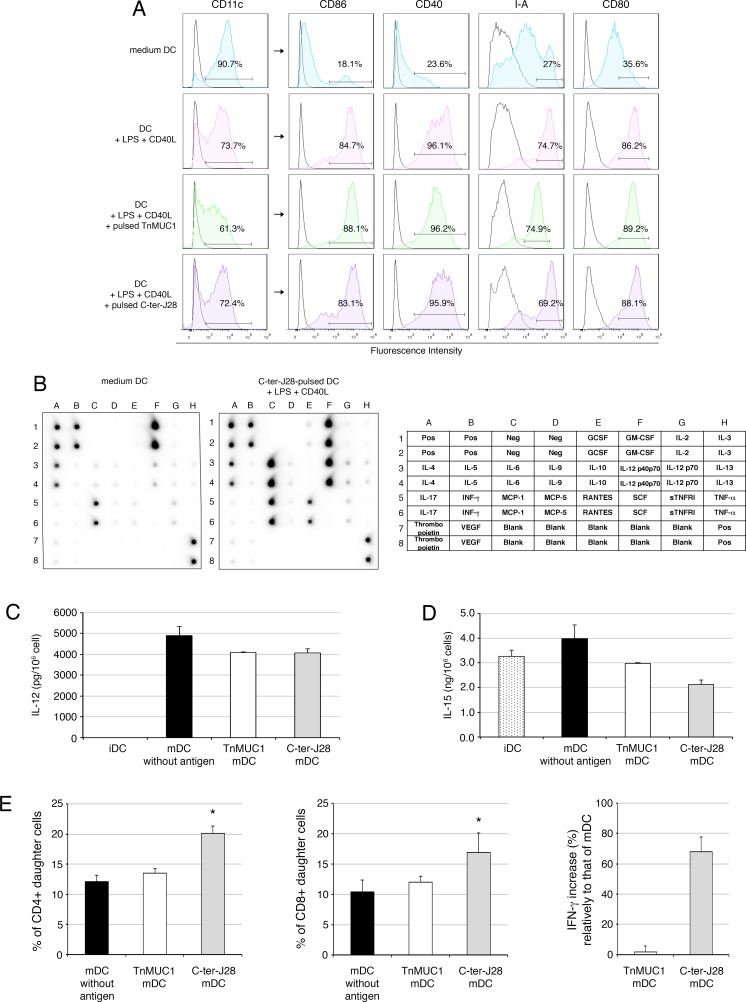
Mature DC pulsed with the glycosylated C-ter-J28^+^ moiety trigger activation of CD3^+^ T-cells from mice immunized with C-ter-J28^+^ **A.** Quality control of DC maturation. At day 5, immature DC (iDC) were pulsed or not with C-ter-J28^+^ or TnMUC1 (at equimolarity, 0.55 μM) for 10 h then cultured with LPS and CD40L for 22 h. The purity of the DC fraction was determined by analyzing CD11c expression. Analysis of cell-surface expression of CD11c, CD86, CD80, CD40, and I-A was performed by flow cytometry. Black histograms represent control isotype and colored histograms staining with anti-CD11c, -CD86, -CD40, -CD80 and -IA. **B.** Protein analysis of cytokines secreted by DC. Culture supernatants from immature DC (medium) and from DC pulsed with C-ter-J28^+^ and matured were collected after 22 h for cytokine detection using cytokine Ab array. Positive spots for GM-CSF and IL-4 were due to the addition of these cytokines in the culture medium. **C.** and **D.** IL-12 and IL-15 production. Culture supernatants were collected after 22 h of maturation for cytokine detection using ELISA assay. Representative results of at least three experiments. **E.** Purified LN CD3^+^ T-cells from mice immunized with C-ter-J28^+^ of pBSDL Caro and CFA were labeled with CFSE. CD3^+^ T-cells were plated at 2.5×10^5^ cells/well in quadruplicate and co-cultured with DC at a ratio of 1 DC: 10 T-cells for 6 days. CFSE dilution was analyzed by flow cytometry and T-cell proliferation evaluated. Percentages of CD4^+^ and CD8^+^ daughter T-cells without addition of DC: 2.5±0.3 (not shown). Representative results of three experiments. Culture supernatants were collected after 24 h for IFN-γ detection. Neither immature nor mature DC alone produced detectable amounts of IFN-γ (not shown). Secretion levels of IFN-γ in co-culture with mDC pulsed with either C-ter-J28^+^ or TnMUC1 were normalized to levels of IFN-γ in co-culture with unpulsed mDC. The results are representative of three independent experiments for C-ter-J28^+^ mDC and two for TnMUC1. (*P < 0.05).

IL-12- and IL-15-secreting C-ter-pulsed mDC or control DC were co-cultured with CD3^+^ T-cells from mice immunized with C-ter-J28^+^. Both CD4^+^ and CD8^+^ T-cell populations proliferated upon encounter with C-ter-pulsed mDC as compared to unpulsed mDC (CD4^+^ T and CD8^+^ T increased by 66 and 62.5 % respectively) and to DC pulsed with the O-glycosylated peptide control TnMUC1 *[*20] (CD4^+^T and CD8^+^T increased by 19.8 and 20 % respectively) (3E). The control TnMUC1 showed no immunoreactivity to mAbJ28 when compared to rC-ter-17R (Supplementary Figure S2). Noticeable cell proliferation with mDC, pulsed or not with TnMUC1, was likely due to their cytokine production. Secretion levels of IFN-γ in co-culture with mDC pulsed with C-ter-J28^+^ or TnMUC1 were normalized to that with unpulsed mDC (257 to 3018 pg/ml depending on the experiment; mean±SD = 1548±1389, *n* = 3). Secretion of IFN-γ in co-culture with C-ter-J28^+^-pulsed mDC was around twice that detected with unpulsed mDC. In contrast, secretion of IFN-γ in co-culture with TnMUC1-mDC was not significantly different to that with mDC.

Thus mDC pulsed with the glycosylated tumor antigen J28^+^ hold potential to activate CD4^+^ and CD8^+^ T-cell responses mandatory to developing adaptive anticancer immunity.

### Immune status of mice injected with tumor antigen-pulsed mDC

Splenocytes freshly collected from recipients vaccinated one to three times showed no significant phenotypic changes from controls (not depicted). However, after 4 days of culture, the percentage of granzyme B-expressing CD4^+^- and CD8^+^ T-cells and NK1.1^+^ cells had conspicuously increased in accordance with C-ter-J28^+^ mDC injection number, by comparison with percentages in unvaccinated mice injected with PBS (4A). Granzyme B-expressing CD4^+^T-cells are indeed reported [21] and may acquire killing activity in various conditions. Interestingly, in co-culture assays, splenocytes from mice DC-vaccinated twice, though not those from naïve controls (4B) or from Panc02-recipients (unpublished data), proliferated with Panc02 (*P* < 0.05) but not with B16-F0 cells. Identical results were obtained at a splenocyte: tumor cell ratio of 25:1. IFN-γ secretion, detectable after 4-day culture, was more pronounced with Panc02 than with B16-F0, and increased with DC-vaccination number. To detect whether C-ter-J28^+^ pulsed mDC could *in vivo* generate C-ter-J28^+^-Panc02-specific cytotoxic activity, splenocytes from the immunized mice served as effectors in the presence of tumor cells. The abnormal round shape of Panc02 occurring upon culture with splenocytes from mice vaccinated with C-ter-J28^+^ pulsed mDC is illustrated in 4C. Also, compared to mDC-recipient mice, splenocytes from C-ter-J28^+^-mDC-vaccinated mice acquired a conspicuous ability to secrete IFN-γ (29 ng/ml versus 5.7 ng/ml) (4D). CD8^+^ T-cells purified from these splenocytes (containing less than 1 % NK1.1^+^, NKp46^+^ cells; not depicted) were able to lyse Panc02 but not B16-F0 cells (4E) while CD8^+^ T-cells from PBS-treated mice were inefficient. Noteworthy, compared to melanoma B16-F0 and B16-F10, Panc02 expressed higher levels of CMH class I required for CTL-mediated lysis in the context of DC-vaccination; however they lacked CD40L, which interacts with DC for cytokine production signaling (unpublished data).

**Figure 4:**
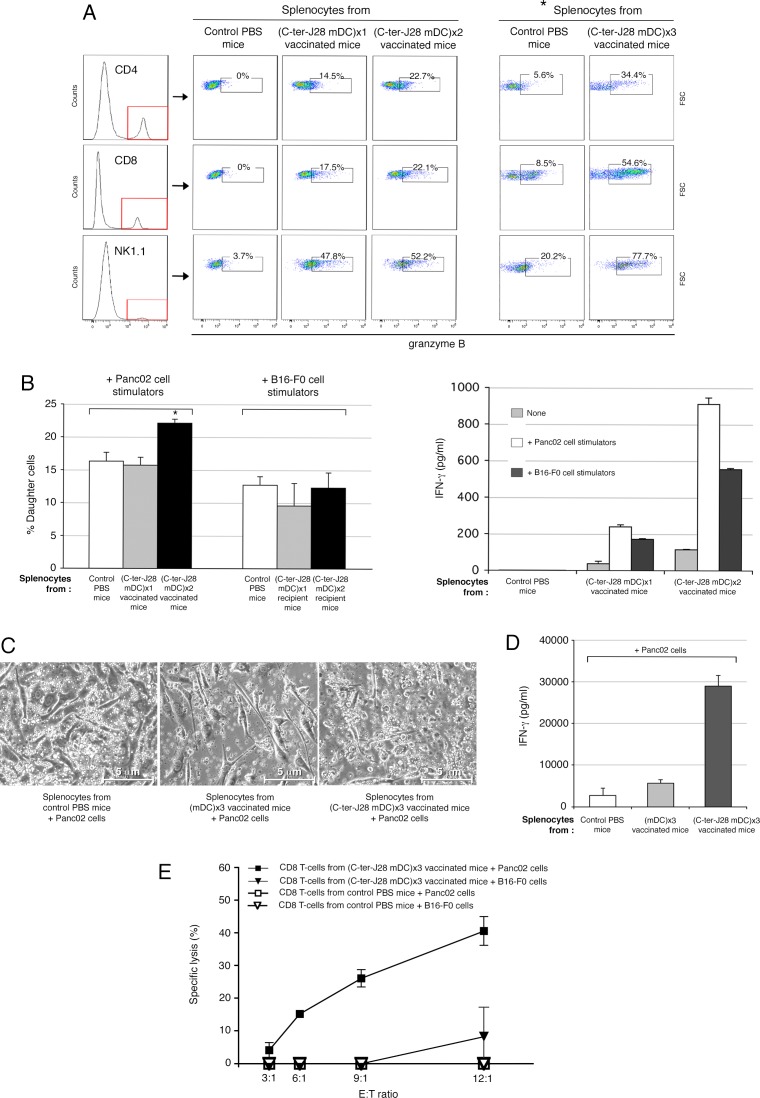
Immune status of C-ter-J28^+^-pulsed mDC-injected mice **A.**-**B.** Immune status of mice injected with tumor antigen-pulsed mDC. C57Bl/6 mice were either vaccinated SC with C-ter-J28^+^-pulsed mDC, once, twice or three times at weekly intervals or received PBS (control mice). **A.** Intracellular expression of granzyme B in CD4^+^, CD8^+^ and NK1.1^+^ cells was determined after 4 days of culture. Splenocytes were collected 5 days after the last DC-injection and cultured in T-cell culture medium; *culture medium was supplemented with IL-2. **B.** Reactivity of splenocytes to mitomycin C-treated Panc02 in co-culture. CFSE-labeled splenocytes were plated with tumor cells at ratio responder:stimulators of 12.5:1. CFSE dilution was analyzed by flow cytometry after 4 days of co-culture and T-cell proliferation evaluated (left panel). Culture supernatants from co-culture were collected after 4 days for IFN-γ detection (right panel). (C-E) C57Bl/6 mice were either vaccinated SC with C-ter-J28^+^-pulsed mDC three times at weekly intervals or received PBS (control mice). Four days following the 3^rd^ DC injection, splenocytes were collected and cultured with mitomycin C-treated Panc02 cells at ratio responder:stimulators of 50:1 with IL-2 for 4 days. **C.** Images of cells after 4 days of co-culture (x200). Co-cultures of splenocytes with tumor cells were examined using a phase-contrast microscope. Panc02 cultured with splenocytes from PBS-or mDC-treated mice exhibit their usual spindled shape; Panc02 cultured with splenocytes from vaccinated mice show dramatic changes in cell morphology and density. **D.** IFN-γ secretion. Culture supernatants from co-cultures were collected after 4 days for IFN-γ detection (mean of two experiments). **E.** Specific cytotoxicity of vaccinated mice CD8^+^ T-cells against Panc02 cells. After co-culture, splenocytes were collected and CD8^+^ T-cells purified before incubation with tumor cells at various ratios for 5 hrs. Supernatants from triplicate or quadruplicate cultures were harvested for determination of LDH activities. (*P < 0.05).

C-ter-J28^+^ DC-vaccinations hence led to enhanced selective cellular immunoreactivity to Panc02, as demonstrated by increased immune cell proliferation and IFN-γ secretion, to increased percentages of granzyme B-expressing CD4^+^ T-cells, CD8^+^ T-cells and NK1.1^+^ cells, and to selective T-cell cytotoxicity.

### Vaccination with C-ter-J28^+^-pulsed-mDC prevents Panc02 tumor development

Motivated by these results, we initiated preclinical trials of tumor DC-vaccination. Control Panc02-recipient mice injected with PBS developed large tumors (mean size of 101.52±45.20 mm^2^) at d35 (5A, left panel). In contrast, three vaccinations with C-ter-J28^+^-pulsed-DC conferred resistance to subsequent Panc02 challenge (*P* < 0.001 at the termination date). Striking protection from tumor development was obtained in 12 mice, 6 of which remained tumor-free indicating the high capacity of DC-vaccination to increase survival. Tumors in the other 6 were smaller (mean: 25.95±33.52 mm^2^ at d35) than those in the unvaccinated Panc02 group (*P* < 0.01). Significant protection was also obtained in the mDC-vaccinated group. To study the effects of DC pulsed with another O-glysolated peptide, we loaded DC with TnMUC1 (25 μg/ml) and injected them using the same protocol as for the C-ter-J28^+^-pulsed mDC. 5A (right panel) shows that C-ter-J28^+^-pulsed mDC induced expected marked protection against Panc02 challenge compared to PBS-controls (*P* < 0.01); the ranges of protection evaluated in mice vaccinated with either mDC or TnMUC1-mDC were similar (*P* < 0.05). A lower concentration of TnMUC1 (5 μg/ml, a concentration equimolar to that of rC-ter-17R-J28^+^) gave the same results. All these findings are illustrated in Supplementary Figure S5A and Supplementary Table S1. Noteworthy was the trend towards a difference in the tumor development curves as a function of time, with that for C-ter-J28^+^-mDC recipients being consistently below those of mDC and TnMUC1-mDC recipients (four experiments). This difference is likely more qualitative than quantitative, an assumption consolidated by the immune status of vaccinated mice with long-term protection (see below).

**Figure 5:**
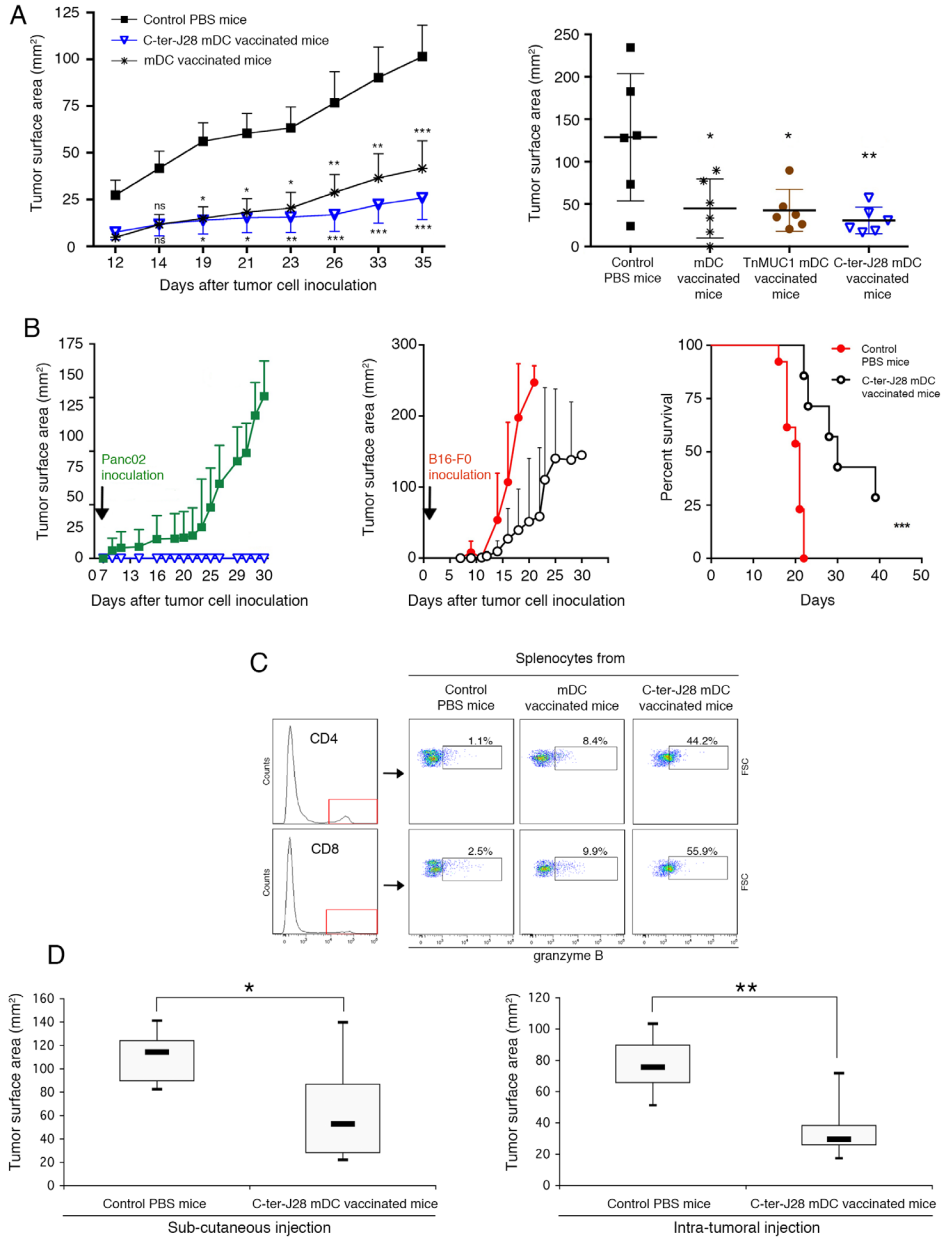
Prophylactic DC-vaccination in Panc02 pancreatic adenocarcinoma model **A.** C57BL/6J mice were either vaccinated SC with C-ter-J28^+^-pulsed mDC, TnMUC1-pulsed mDC or mDC three times at weekly intervals or they received PBS (control mice). Four days following the 3^rd^ DC-injection, mice were challenged SC with Panc02 in the contralateral flank. Left panel: experiments performed twice on groups of six mice gave similar results (pooled experiments designed experiment I). Data are expressed as mean tumor surface area ± SEM. Six of the 12 mice vaccinated with C-ter-J28^+^ mDC remained free of tumors. Comparisons between groups were made by two way-ANOVA repeated measurements, and differences were considered significant at *P* < 0.05 (*P < 0.05, ** *P* < 0.01, *** *P* < 0.001). Right panel: *n* = 6 per group. DC were loaded with C-ter-J28^+^ or TnMUC1 (25 μg/ml, experiment II; 5 μg/ml experiment III in Supplementary Figure S5). *P < 0.05, ** *P* < 0.01; Holm-Sidak's post-hoc test. Long-term protection provided by prophylactic DC-vaccination. **B.** Mice receiving prophylactic DC-vaccination and remaining tumor-free were challenged after 43 (experiment I) or 35 (experiment III) days with Panc02 (left panel, open blue triangle, *n* = 7) or B16-F0 cells (middle panel; open black circle, *n* = 7) and compared against their respective controls injected with Panc02 (filled green square, *n* = 9) and B16-F0 (filled red circle; *n* = 13). Data pooled from experiments I and II are expressed as mean of tumor surface area ± SEM. Graph (right panel) shows a Kaplan-Meier survival curve from mice that received PBS (controls; filled red circle; *n* = 13 ) or Cter-J28^+^-mDC (filled black triangle, *n* = 7) followed by B16-F0 inoculation. ***P = 0.0002; log-rang test. **C.** Fifty-nine days after the second challenge with Panc02 (experiment I), splenocytes were collected and expression of granzyme B was determined on the same day (Day 0) without further culture. Therapeutic vaccination. **D.** Fourteen days after Panc02 cell challenge (once a palpable nodule had formed), mice were injected SC (left panel) or intratumorally (right panel) with C-ter-J28^+^-pulsed mDC three times at weekly intervals, or they received PBS (control mice; *n* = 6). Measurements at day 13 were subtracted from those at day 31 (termination date). Mann-Whitney test was used to compare tumor development of C-ter-J28^+^-pulsed mDC recipient mice with that of control mice (*P < 0.05, ** *P* < 0.01).

Tumor protection in C-ter-J28^+^ mDC-vaccinated mice, shown in the experiment depicted in 5A (left panel), correlated with a high level of spontaneous proliferation and IFN-γ secretion, both dependent on splenocyte concentration (Supplementary Figure S3 A and B). Thus C-ter-J28^+^ DC-vaccinations induced an IFN-γ signature in spleen. Tumor protection was also associated with differentiation of CD4^+^ and CD8^+^T-subpopulations and NK1.1^+^activation as measured by granzyme B expression, not detected in the control conditions at day 35 after tumor challenge (Supplementary Figure S3C). At day 28 after tumor challenge, among protected DC-vaccinated mice, CD4^+^T-subpopulation expressing granzyme B in C-ter-J28^+^DC recipient splenocytes had increased compared to that in mDC- and TnMUC1-mDC recipient splenocytes, respectively by 69% and 77% (Figure S5B). All groups receiving mDC pulsed or not displayed similar high percentages of NK1.1^+^expressing granzyme B.

### Vaccination with C-ter-J28^+^-pulsed-mDC establishes long-term protection against Panc02-induced tumor development

The mice fully protected against tumor development (depicted in 5A) were rechallenged with either Panc02 or melanoma B16-F0, 43 days after the first inoculation. Whereas controls inoculated with Panc02 developed large tumors (mean 128.8±38 mm^2^ at d30), vaccinated mice showed long-term survival without tumors. Interestingly upon challenge with melanoma B16-F0 (33-54 days later; 2 experiments) 3 out of 4 of these Panc02-resistant mice developed tumors similarly to the 10 control melanoma-recipients and died; one mouse remained tumor-free until the termination date. Differences between groups however were not significant due to insufficient statistical power (Fisher's exact test; *P* = 0.143). At euthanasia, the C-ter-J28^+^-mDC-vaccinated recipients challenged with Panc02 displayed high expansion of CD4^+^- and CD8^+^-T-cells expressing granzyme B (more than 44%) in spleen compared to mDC-recipients (less than 10%) and a PBS-control mouse with late tumor development (2.5%) (5C). Tumor cell challenges had no impact on immune status, as confirmed by the negligible or low level of cell activation observed in splenocytes from mice injected with Panc02 or B16-F0 (Supplementary Figure S4).

To strengthen these results, another experiment was performed with six 17R-C-ter-J28^+^ mDC-vaccinated mice receiving either B16-F0 or Panc02. B16-F0 administration led to the development of melanoma in all three rC-ter-17R-J28^+^ mDC-vaccinated mice, with tumor surface areas of 61.5, 143 and 250 mm^2^ (surface area mean±SD = 151± 90 mm^2^) compared to a mean surface area of 250 mm^2^ - the limit we fixed in the experimental procedure - in the three B16-F0 controls. In contrast, all three rC-ter-17R-J28^+^ mDC-vaccinated mice showed complete resistance to Panc02 while the three non vaccinated Panc02 controls developed tumors with a mean surface area of 56 ± 8 mm^2^ at day 28. All the results of the experiments presented above were pooled (5B). To resume, one sub-group of C-ter-J28^+^-mDC vaccinated mice fully resisted a second challenge by Panc02, while the other sub-group challenged instead with B16-F0 displayed only a slight delay in melanoma development and most did not survive.

### Vaccination with C-ter-J28^+^-pulsed-mDC treats established tumors

Immunotherapy efficiency was demonstrated by the reduced size of tumors in vaccinated mice compared to those in PBS-treated controls (mean tumor size of 52.8 mm^2^ in SC DC-vaccinated *vs* 114.3 mm^2^ in controls; *P* < 0.01; and 29.5 mm^2^ in intratumorally DC-vaccinated *vs* 75.6 mm^2^ in controls; *P* < 0.05) (5D). Thus, intratumoral DC-injection was more effective than the SC route.

### Vaccination with C-ter-J28^+^-pulsed-mDC attenuates deleterious effects of pancreatic tumor

During the MRI follow-up, no sign of cachexia could be detected in vaccinated or non-vaccinated mice. Primary pancreatic tumors appeared solid with well-defined margins at early time points on MRI, and mixed solid/cystic at later time points. Longitudinal MRI indicated a trend towards lower tumor volume in DC-vaccinated mice (Supplementary Figure S6A). However due to pleomorphic multi-lobulated appearance, volumetry of the primary tumor at d17-18 was only an estimate and only 3 out of the 6 tumors in each group were considered measurable. 6A-6E shows representative images of the primary tumor and its spread within and beyond the peritoneal cavity in two non-vaccinated mice in contrast to limited peritoneal spread in a vaccinated mouse (details in figure legend). Significant effects of DC-vaccination were observed on the MRI-based disease progression score (Supplementary Table S2 and S3), with vaccinated mice showing significantly lower scores than non-vaccinated mice at d17-18 (disease progression score of 7.5±1.18 in vaccinated mice [*n* = 6] and 11±0.58 in non-vaccinated mice [*n* = 6]; two-way ANOVA F2,30 = 0.7151, *P* = 0.4973 for interaction; F1,30 = 9.694, *P* = 0.0040 for treatment effect; F2,30 = 27.29, *P* < 0.0001 for time effect; Bonferroni post hoc test *P < 0.05 for group comparison at d17-18) (6F). MRI showed that despite direct extension to peripancreatic tissues (retroperitoneal and mesenteric fat, peritoneum) and often unresectable tumor observed in both groups, the C-ter-J28^+^-DC-vaccine delayed or prevented metastasis (distant lymph nodes, liver, kidneys and lungs) and reduced secondary abdominal disease (organ displacement, splenomegaly, obstructed bile ducts and distended gall bladder) (6G, supplementary Figure S3B). Pleural effusion and pleural metastasis were detected at d17-18 only in non-vaccinated mice and resulted in rapid death after anesthesia induction. Thus, vaccination with C-ter-J28^+^-pulsed DC hampered growth, invasiveness and metastasis of pancreatic tumors.

**Figure 6:**
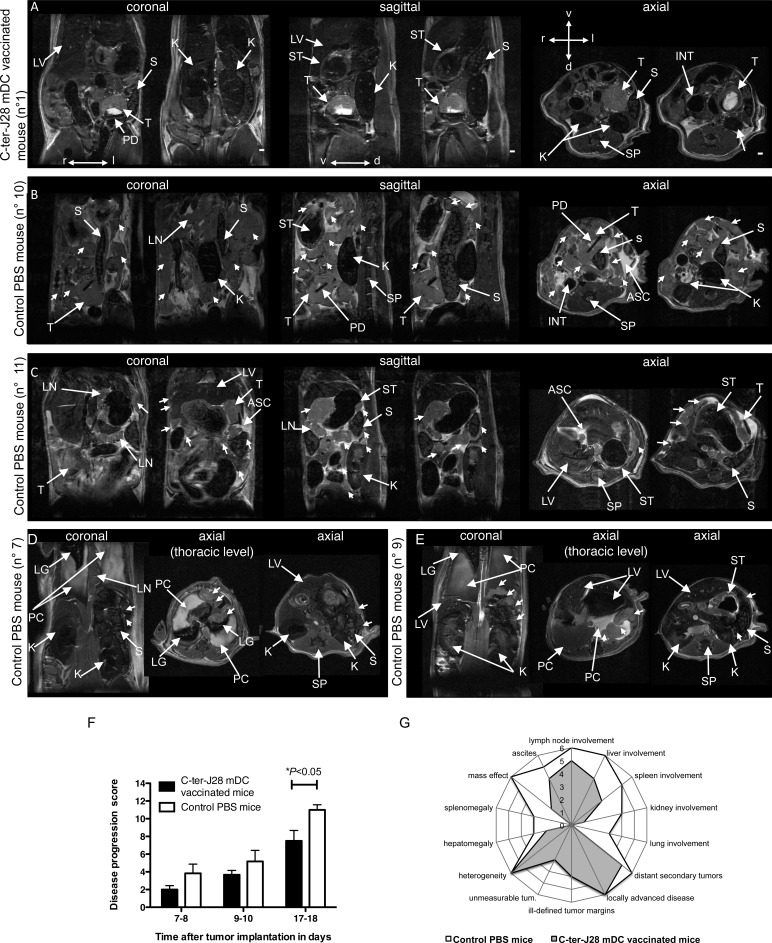
C-ter-J28^+^-mDC vaccination attenuates deleterious effects of orthotopically implanted pancreatic adenocarcinoma cell induced-tumor monitored by abdominal MRI at d17-18 **A.** C-ter-J28^+^-DC-vaccinated mouse (score: 3) with a well-marginated primary tumor. **B.**-**E.** Control PBS mice. Mouse (score: 13) with a lobulated primary tumor, numerous contiguous and distant tumors (arrowheads), swollen LNs, splenomegaly, kidney and spleen displacement, and ascites. Ascites hyperintensity is probably linked to a high protein-content in the peritoneal fluid. **C.** Mouse (score: 10) showing swollen LNs, multiple local and distant tumors (arrowheads), tumor tissue enveloping the spleen, metastasis of the kidney (mass below the kidney capsule), and an abnormal nodular aspect of the liver (axial image). **D.** Mouse (score: 11) with pleural effusion, lung compression by pleural fluid, metastatic mediastinal LNs (thoracic axial image, arrowheads), spleen compression by surrounding tumor tissue, and SC tumors (arrowheads, axial image). **E.** Mouse (score: 12) with bilateral pleural effusion, pleural metastases in the left cavity (coronal and thoracic axial images, arrowheads), and in the stomach wall. Note the presence of a tumor between the spleen and the kidney (axial image, arrowhead). **F.** MRI-based disease progression score. Significant differences were observed between vaccinated and non-vaccinated mice at d17-18 (*P < 0.05). **G.** Radar chart comparing the total sum of each MRI feature of the score between vaccinated and non-vaccinated mice at d17-18. Abbreviations: d, dorsal; l, left; r, right; v, ventral; ASC, ascites; INT, intestine; K, kidney; LG, lungs; LV, liver; LN, lymph nodes; PC, pleural cavity; PD, paper disc; S, spleen; SP, spine; ST, stomach; T, primary tumor. Scale bars: 1.

### Source of BSDL-J28^+^

Considering the initial difficulties faced recovering substantial amounts of purified recombinant C-ter-J28^+^, we turned our attention towards bile salt-stimulated lipase (BSSL) present in human milk, which differs from BSDL only in glycosylation pattern [22]. We showed that BSSLs were both immunoreactive to mAbJ28 and immunogenic (Supplementary Figure S7).

## DISCUSSION

We have validated pancreatic tumor glycoepitope C-ter-J28^+^ as a good inducer of anticancer adaptive immunity in mice and so exemplified a proof-of-principle study from antigen discovery as a tumor marker in human tissue to immunogenic target for DC-vaccination in murine pancreatic cancer. Our data provide new strong evidence that (i) fucose-rich epitopes of pBSDL-J28^+^, appearing during human pancreatic oncogenesis processes, are also expressed by murine PDAC cells Panc02; (ii) DC pulsed with the tumor pancreatic antigen skew adaptive immunity towards Th1 polarized responses; (iii) vaccination with C-ter-J28^+^ DC meets cancer vaccine objectives by inducing anti-Panc02 CD8^+^ T-cells and NK cells required to eradicate tumors and by promoting long-lasting resistance [23].

We first molecular identified the PDAC-specific antigen by demonstrating the presence on C-ter-J28^+^ of a glycosylation-dependent immunodominant epitope, common to various pBSDLs, that is able to trigger adaptive immune responses. Our findings contradict the hypothesis that the reported poor immunogenicity of Panc02 tumor could be due to lack of recognition of such modified auto-antigens. Rather, low tumor antigen expression, lack of co-stimulatory signals and other factors may be partly responsible [4].

A critical step in vaccination is the efficient presentation of cancer antigens to T cells [4]. We therefore investigated the potential of C-ter-J28^+^-pulsed mDC to activate CD4^+^ and CD8^+^ T-cell responses mandatory to develop adaptive anticancer immunity. We found that DC, high producers of two cytokines important for both innate and adaptive immunities, IL-12 and IL-15 [24, 25], when loaded with glycoantigen-J28^+^, drove CD3^+^ T-cells toward Th1 polarization with expansion of antigen–specific CD4^+^ and CD8^+^ T-cells secreting IFN-γ. These findings agree with the known ability of IL-12 to induce antigen-specific immunity by inducing or increasing Th1 cell and CTL responses [24]. IL-12 induces T-cells and NK cells to produce cytokines, particularly IFN-γ with which it likely cooperates to induce T-cell clones that expand in response to specific antigens and differentiate into Th1 cells. Our data also support evidence that the other T-cell and NK cell activating factor IL-15 may skew naive CD8^+^ T-cell differentiation towards effectors secreting type 1 cytokines [26].

Importantly, *in vivo*, tumor antigen J28^+^-pulsed mDC were able to promote Th1 and NK responses in vaccinated mice, monitored by a T-cell proliferative response to epitope J28^+^-Panc02 cells, secretion of IFN-γ, increased % of CD4^+^T-, CD8^+^T- and NK1.1-cells harboring granzyme B, and CTL-lysis restricted to PDAC cells. Sustained secretion of IFN-γ was the hallmark of splenocytes from C-ter-J28^+^-DC-vaccinated mice in response to Panc02 in culture, with the number of C-ter-J28^+^-DC-vaccinations impacting the IFN-γ secretion level. A moderate secretion was obtained in response to B16-F0, likely from cells other than CD4^+^- and CD8^+^-T, such as activated NK and natural killer DC [26, 27]. These distinct responses reflect T-cell specificity towards antigen-bearing Panc02, as reported for *in vitro* TAA-specific anti-melanoma CTL responses elicited by TAA-loaded DC vaccination [28].

C-ter-J28^+^ antigen-pulsed-DC vaccination prevents ectopic growth of Panc02, in line with previous reports using different preparations of Panc02. In prophylactic conditions, increased survival was obtained in mice vaccinated with mDC pulsed with Panc02 heat-treated lysate [29] and irradiated Panc02 [30]. In therapeutic conditions, Panc02-RNA transfected-DC injection into orthotopic tumors induced their regression [30]. While these preclinical trials show *in vitro* [29, 31] and *in vivo* [30] that loaded DC activate CTLs, none has controlled the specificity of vaccination by using target tumor cells from other cancers. As long as the tumor antigens from Panc02 remain undefined, the observed immunity may target surface antigens common to tumor cell lines from various origins. In our test of long-term protection, we demonstrated that C-ter-J28^+^-pulsed-DC-vaccinated mice, fully protected against Panc02-tumor development, all resisted a second Panc02 challenge while most did not survive melanoma challenge. This could be attributed to memory CD4^+^ and CD8^+^ T-cells, consistent with the ability of IL-15 yielded by DC to induce increased percentages of effector memory CD8^+^ T-cells [25, 32]. As IL-15 also impacts on memory NK cells [33], one cannot exclude their possible role. Yet, since C-ter-J28^+^-mDC-vaccination resulted in an at least 5-fold increase in the percentage of CD4^+^- and CD8^+^-T-cells expressing granzyme B in splenocytes of recipients by comparison to mDC-vaccination, one can reasonably assume that CD4^+^ and CD8^+^ T-cell activation is associated to the long-lasting protection induced by Ag-pulsed-DC. Moreover, recent findings underscore the essential role of IL-12 produced by patients with melanoma after DC vaccination in the development of therapeutic antigen-specific CD8^+^ T-cell immunity [34]. Overall, these results suggest the involvement of tumor-specific CTL at least in long-term resistance to Panc02-tumor development, induced by tumor antigen-J28^+^-pulsed mDC.

Immunization with antigen-pulsed mature DCs, which aims to elicit antitumor T cell responses, may in fact also lead to significant antitumor immunity in an NK cell-dependent manner, reflecting interplay between DC, NK and CD4^+^ and/or CD8^+^ T-cells as reported in melanoma and other tumor models [35, 36]. Such NK involvement is supported by the finding of an increased percentage of NK1.1^+^ cells expressing granzyme B in mice receiving unloaded mDC or mDC loaded with the O-glycosylated polypeptide control, TnMUC1.

Protective CTL responses requiring a contribution of both CD4^+^ T helper cells and NK cells are reported after vaccinations, though only in peculiar conditions, with TAA gene-modified DC [37] or surrogate antigen-pulsed DC [38]. Protection depending on TAA-specific antitumor CTL responses elicited *in vivo* by TAA-loaded DC has, to our knowledge, never as yet been reported. To summarize, C-ter-J28^+^ DC-vaccinations led to an immune status characterized by expansion of anti-C-ter-J28^+^-tumor CD8^+^ T-cells, which *in vitro* specifically recognize Panc02 and kill them, and to NK cell activation. Further studies are now needed to delineate the *in vivo* relative mechanistic contribution of CTL, CD4^+^ T-cells and NK cells.

Furthermore, in control experiments using unloaded mDC-immunization, we and others [30, 38-40] have found some degree of protection against tumor challenge, which is in line with the demonstration that the unloaded mDC may induce NK cell-mediated immunity [38-40]. We obtained similar results using the O-glycosylated polypeptide control, TnMUC1, for mDC pulsing. This correlates in particular with our mDC producing IL-12, known to enhance NK cell cytotoxicity [24]. The ability of unloaded DC to exert protection against tumor challenge, to prevent the development of tumor metastases and to establish long-term survival is proved to be dependent on NK cells, mainly in melanoma models [40, 41].

Therapeutically, intratumoral immunization provided a more potent protective immunity than SC immunization, as reported by others [31, 42], but was less efficient than prophylactic vaccination [31]. Improving vaccine efficiency via their combination with agents targeting different pathways is thus required [4]. Such combinations might synergistically generate more potent immune responses by activating DC and so fully exploiting their capacity to trigger anticancer responses.

Lastly, in an orthotopic model of pancreatic cancer, non-invasive longitudinal serial MRI confirmed that disease progression in the murine model is similar to clinically encountered adenocarcinomas [43]. Although both groups of mice presented with locally advanced disease, metastasis was delayed or prevented by the C-ter-J28^+^-DC-vaccine, and secondary abdominal disease was reduced. Genetically-engineered mouse models (*Pdx-1-Cre/Kras*^*G12D*^*/Ink4a/Arf*^*lox/lox*^ and *Pdx-1-Cre; LSL-Kras*^*GD12*^*; LSL-Trp53*^*R172H*^ mice), which develop pancreatic tumors [44, 45], could not be used since the tested tumors showed no reactivity to mAbJ28. This might be due to the fact that in addition to these three genes showing high frequency of mutations, a great number ( > 50 in average) of gene mutations is found in human PDAC [46]. They affect at least a dozen key signaling pathways. Their direct or indirect consequences might therefore be responsible for the observed human pathological features such as hyperfucosylation of pBSDL.

In the proposed model, Panc02-cell-induced pancreatic tumor was associated with poorly developed stroma and caused neither the typical ductal lesions seen in human PDAC nor those found in the genetically engineered mice. Interestingly, it induced peritoneal carcinomatosis, commonly observed in patients suffering from PDAC [47] and considerably contributing to their demise [48], and metastasis. This model is widely explored for the development of novel therapeutic strategies, and here essentially provides relevant targets for tumor antigen-DC vaccination.

The main limitation of our model concerns the source of C-ter-J28^+^, more precisely the impossibility of obtaining pathological pancreatic juices from patients and the initial inefficiency of recombinant C-ter-J28^+^ production. Yet, this production has today greatly improved. We have also presented one potential way of circumventing this problem by detecting the glycotope-J28 within BSSL from human milk.

To conclude, our findings provide new insights into the immunogenicity of glycosylated pancreatic TAA, and for the first time demonstrate in a preclinical model that the selective and spontaneous expression of TAA such as pBSDL-J28^+^ on PDAC cells and tissues makes them pertinent targets of DC-immunotherapy. C-ter-J28^+^ DC-vaccination could represent a novel option for PDAC multiple adjuvant therapy in humans [for review see 49].

## MATERIALS AND METHODS

### Ethics statement

The investigation was conducted in accordance with the French guidelines for animal care and the directive 2010/63/EU of the European Parliament, and was approved by the local ethics committee of Aix-Marseille University.

### Mice

Seven to 10 week-old C57BL/6J Rj (H-2^b^) mice and NMRI-nu *(nu/nu)* mice were from Janvier (Le Genest-St. Isle, France).

### Cell lines

Cell lines derived from C57BL/6 included the highly tumorigenic murine pancreatic carcinoma cell line with ductal morphology, Panc02, kindly provided by Dr V. Schmitz and E. Raskopf (University of Bonn, Germany), and the metastatic clones of the B16 melanoma, B16-F0 and B16-F10. Also used were HEK-293T cells isolated from human embryonic kidney and modified with the SV40 Large T-antigen. HEK-293T cells were transfected with the pSecTag-2B plasmid (Life Technologies, Saint Aubin, France) encoding for hexahistidine-tagged 6 repeated sequences of the human C-terminal domain (C-ter-6R) of BSDL cDNA and thus called HEK-C-ter-6R, or for the BSDL cDNA comprising 17 repeated sequences of the human C-terminal domain and thus called HEK-BSDL-17R.

Before injection, cells were tested negative for mycoplasma contamination.

### Antigens

pBSDLs-J28^+^ were purified from pancreatic juices of patients suffering from PDAC [11] and the pBSDL-J28^+^ C-terminal glycopolypeptide (C-ter-J28^+^) was then obtained by cyanogen bromide cleavage of pBSDL-J28^+^ [14]. These C-ter-J28^+^ were used in all the experiments unless indicated. Recombinant BSDL-17R-J28^+^ (rBSDL-17R-J28^+^) was purified from HEK-BSDL-17R cell culture supernatant and its C-terminal glycopolypeptide (rC-ter-17R-J28^+^) obtained as mentioned above. EAT (EATPVPPTGDS) and GAP (GAPPVPPTGDS) peptides, and the synthetic pBSDL C-terminal polypeptide (77 mers), were purchased from Proteogenix (Oberhausbergen, France). The synthetic MUC1 polypeptide (100 mers) represents five repeats of 20 amino acids, O-glycosylated *in vitro* with GalNac incorporated within 3-5 threonines per repeat [20]. This glycosylated peptide, designated TnMUC1, was kindly provided by Pr O. Finn (Univ of Pittsburgh School of Medicine, PA, USA). Purified bile salt-stimulated lipases (BSSL) were a generous gift of Pr O. Hernell (Umea University, Sweden).

### Tumor induction and vaccination strategies

Panc02 (5×10^5^to 1×10^6^) and B16-F0 (1 to 3×10^5^) were inoculated subcutaneously (SC) into the flank of C57BL/6 mice and HEK-C-ter-6R or HEK-293T (4×10^6^) into the flank of nude mice. Tumor growth was expressed as the product of perpendicular diameters, measured using a digital caliper. Mice were sacrificed when tumors exceeded 250 mm^2^. For orthotopic conditions, cubes (2 mm^3^) of SC Panc02-tumors were surgically transplanted onto the pancreas (see a novel procedure detailed below). For prophylactic vaccination, DC were injected SC into one flank, three times at weekly intervals unless indicated. For therapeutic vaccination, DC were injected SC or intratumorally when tumors had formed a palpable nodule.

### Orthotopic tumor implantation

The pancreas of anesthetized mice was exposed after laparotomy on a sterile gauze. A cube (2 mm^3^) of a SC tumor induced by pancreatic adenocarcinoma Panc02 cells, was placed on a sterile disc (diameter: 2 mm) of Whatman paper (N°3); a drop of Histoacryl® glue (Brown, Melsungen, Germany) was put next to the tumor. The paper was then stuck onto the pancreas with the tumor in between. After suturing, mice received SC injections of analgesic (Buprenorphine 0.1 mg/kg) after the operation and again a few hours later.

### Antibodies (Ab)

Control isotype Abs from mouse and rabbit were purchased from BD Pharmingen and Beckman Coulter, respectively. Polyclonal (p)AbL64 and pAbL32 Abs, directed against a mixture of human BSDL and pBSDL from pathological pancreatic juices, were produced in our laboratory. The mAbJ28 was a gift from Dr. M. J. Escribano (Inserm U260, Marseille, France). Anti-CD4-A647, anti-CD8-PE, anti-IA-A647, and anti-MHC-I (H-2K^b^)-PE Abs were from Ozyme (St Quentin-en-Yvelines, France); anti-CD11c-eFluor450, anti-granzyme B-PB, and anti-IFN-γ-FITC from Ebioscience (San Diego, CA); anti-CD40, anti-CD80 and anti-CD86 from BD Biosciences (Le Pont-de-Claix, France); anti-CD40L-FITC from Proteogenix (Oberhausbergen, France); and anti-NK1.1-A647 and anti-NKp46-PE Abs from Miltenyi Biotec (Bergisch Gladbach, Germany).

### Flow cytometry

Cells were labeled as described in [12] using 2 or 4% paraformaldehyde fixation. For intracellular staining, cells were incubated for 4 hours at 37°C with monensin (GolgiStop, BD Pharmingen, San Diego, CA). After Fc receptor blocking using anti-CD16/CD32 (BD Pharmingen) and staining with anti-CD4, anti-CD8 and anti-NK1.1 Abs, intracellular IFN-γ and granzyme B production was determined using FoxP3/Transcription Factor Staining Buffer Set (Ebioscience, San Diego, CA). Fluorescence was quantified using a Gallios flow cytometer (Beckman Coulter, Roissy CDG, France). Data were analyzed using FlowJo software (Tree Star, San Carlos, CA). The results are expressed as percentages of fluorescent cells stainted by specific Ab minus percentages of fluorescent cells stained by isotype Ab.

### Immunohistocytochemistry

Immunohistocytochemistry was performed using the Dako ARK^TM^(Dako, Hamburg, Germany) according to the manufacturer's instructions. The staining was completed by incubation with substrate-chromogen 3,3-diaminobenzidine (DAB). Sections were counter-stained with hematoxylin-phloxin.

### T-cell proliferation

Nine days after immunization, a cell suspension was prepared from draining lymph nodes (LN). T cells were purified using a negative isolation kit (Invitrogen) when indicated. After labeling with carboxyfluorescein succinimidyl ester (CFSE) (1μM, Invitrogen), cells were plated at 2-4 × 10^5^ cells/well in culture medium [RPMI-1640, 5% fetal bovine serum (FBS), 50μM β-mercaptoethanol, 1% non-essential amino acids (Invitrogen) and <1% sodium pyruvate (Invitrogen)] containing antigens. Cultures were carried out in triplicates or quadruplicates.

### Generation of dendritic cells (DC), DC-antigen loading and maturation

DC were generated from C57Bl/6 mouse bone marrow according to Inaba's protocol [50], and cultured with 20 ng/ml of GM-CSF (Immunotools, Friesoythe, Germany*).* At days 2 and 4, supernatant was removed and replenished with fresh DC media. IL-4 (20 ng/ml) was added when indicated. At days 5-6, DC were loaded with antigens. Maturation was induced with a combination of LPS (0.1μg/ml; Sigma-Aldrich) and 3T3 murine fibroblasts transfected with murine CD40L at 1/5 ratio (3T3/DC). At days 6-7, expression of surface membrane markers was controlled by cytometry analysis.

### Cytokine detection in DC-culture supernatants

Culture supernatants were collected for cytokine detection by ELISA (for IL-12, Ozyme; for IL-15, Raybiotech, Inc. Norcross, GA) and by RayBio® Mouse Cytokine Antibody Array I (RayBiotech), before being revealed using Gbox system (GeneSnap software, Syngene, Ozyme).

### Cytotoxic assays

Cytotoxicity was assessed by lactate dehydrogenase (LDH) release assay using the CytoTox 96 Nonradioactive Cytotoxicity kit (Promega Corporation, Madison, WI, USA) following the manufacturer's protocol.

### Abdominal MRI

DC-induced response was assessed *in vivo* on days 7-8, 9-10 and 17 or 18 after orthotopic tumor implantation by serial MRI. Twelve tumor-bearing and two control mice were imaged at very high field on an 11.75 T vertical Bruker AVANCE 500 WB wide-bore MR system (Bruker, Ettlingen, Germany) [51], with a transmitter/receiver volume birdcage coil (diameter 30 mm), under gaseous anesthesia (1-1.5% isoflurane in air). The respiratory rate was kept at 70±20 breaths per minute (bpm) and monitored using a pneumatic pressure probe and an MRI compatible monitoring and gating system (PC-sam, Small Animal Instruments Inc., Stony Brook, NY). Body temperature was maintained at 37°C using the magnet gradients. Multi-slice images were acquired, 15 to 60 minutes after intraperitoneal injection of 80 μl of 0.5 M gadoteric acid (DOTAREM®, Guerbet, Villepinte, France), in the sagittal, coronal and transverse planes using a 2D spin-echo sequence (repetition time, 448 ms; echo time, 14 ms; flip angle, 50°; 2 accumulations) with respiratory gating to reduce motion artefacts. Geometrical parameters were as follows: matrix, 300 × 240 for sagittal and coronal planes and 240 × 240 for the axial plane; field of view, 24 × 30 mm for sagittal and coronal planes and 24 × 24 mm for the axial plane; spatial resolution, 100 × 100 × 500 μm^3^, 20 contiguous slices. Total acquisition time was 8 to 12 minutes per plane depending on the respiration rate. Tumor growth, morphology and spread were evaluated by two trained MRI scientists (ATP-B and AV) blinded to the treatment conditions on 20 contiguous slices in three orthogonal planes covering the entire primary tumor, using manual volumetry and a disease progression score inspired by the Tumor-Node-Metastasis (TNM) staging system [43].

### Statistical analysis

The statistical analysis was performed using the two-way analysis of variance (ANOVA) test followed by a Bonferroni test, and the Mann-Whitney, the Kruskall-Wallis and the Fisher's exact tests. Values are reported as means ± SEM. Values of *P* < 0.05 were considered significant; *P* < 0.1, borderline significant. The survival curves were determined using the Kaplan-Meier method. The log-rank test was used to compare curves between study and control groups. Principal component analysis (PCA) on variances of MRI parameters was performed with JMP 9 software.

## SUPPLEMENTARY MATERIAL FIGURES AND TABLES



## Abbreviations

BSDL: bile salt-dependent lipase
C-ter: C-terminal moiety
DC: dendritic cells
iDC: immature DC
IP: intraperitoneally
mDC: mature DC
mABJ28: monoclonal antibody J28
PDAC: pancreatic ductal adenocarcinoma
SC: subcutaneously
TAAs: tumor-associated-carbohydrate antigens
