# Supplementary material for: A pancreatic tumor-specific biomarker characterized in humans and mice as an immunogenic onco-glycoprotein is efficient in dendritic cell vaccination
Source: Oncotarget. 2015 Jun 8;6(27):23462–79. doi: 10.18632/oncotarget.4359 (PMC4695130; doi:10.18632/oncotarget.4359)
Supplement: Supplementary file 1 [file oncotarget-06-23462-s001.pdf]

A pancreatic tumor-specific biomarker characterized in humans and mice as an immunogenic onco-glycoprotein is efficient in dendritic cell vaccination

Supplementary Material

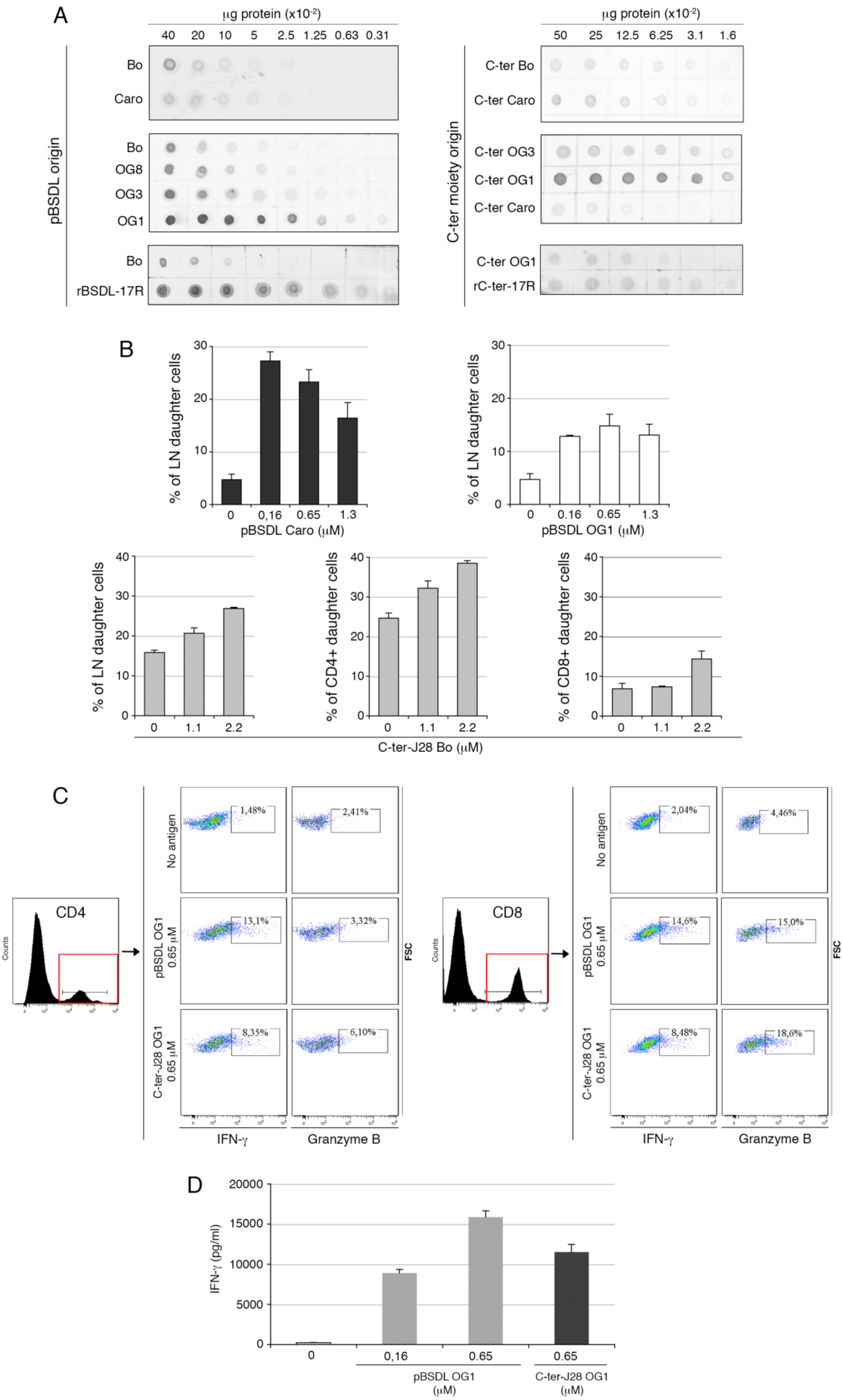
A
µg protein (x10-2)
40 20 10 5 2.5 1.25 0.63 0.31
Bo
Caro
Bo
OG8
OG3
OG1
Bo
rBSDL-17R
pBSDL origin
µg protein (x10-2)
50 25 12.5 6.25 3.1 1.6
C-ter Bo
C-ter Caro
C-ter OG3
C-ter OG1
C-ter Caro
C-ter OG1
rC-ter-17R
C-ter moiety origin
B
% of LN daughter cells
pBSDL Caro (µM)
0 0,16 0.65 1.3
pBSDL OG1 (µM)
% of LN daughter cells
% of CD4+ daughter cells
% of CD8+ daughter cells
0 1.1 2.2
C-ter-J28 Bo (µM)
C
CD4
Counts
No antigen
pBSDL OG1 0.65 µM
C-ter-J28 OG1 0.65 µM
1,48%
2,41%
13,1%
3,32%
8,35%
6,10%
FSC
IFN-γ
Granzyme B
CD8
Counts
2,04%
4,46%
14,6%
15,0%
8,48%
18,6%
D
IFN-γ (pg/ml)
20000
15000
10000
5000
0
0 0,16 0.65 0.65
pBSDL OG1 (µM)
C-ter-J28 OG1 (µM)

Supplementary figure S1: Full-length pBSDLs from different patients with PDAC and C-ter-J28 moieties induced *in vitro* activation of $CD4^+$ and $CD8^+$ T-cells from LN from mice immunized with pBSDL. (A) Level of J28 expression in pBSDLs from different patients with PDAC and their corresponding C-ter, and in rC-ter-17R-$J28^+$ evidenced by dot blot dilution. Dilutions were made from 0.4 mg/ml for pBSDL and 1mg/ml for C-ter. One microliter was spotted onto a nitrocellulose membrane. Membranes were incubated with mAbJ28 and then by alkaline phosphatase-labeled secondary Ab (Sigma, St Louis, MO). (B) T-cells from mice immunized with full-length pBSDL proliferated in the presence of various pBSDLs or of the glycosylated C-ter-J28. Isofluorane-anesthetized C57BL/6J mice (12 weeks old) were immunized at the base of the tail with 0.2 ml of emulsion containing 50 to 100µg of pBSDL-Caro in PBS and incomplete Freund's adjuvant supplemented with *Mycobacterium tuberculosis* (5mg/ml). They received 300 ng of *Pertussis* toxin (Pt) (Sigma-Aldrich) IP, before the immunization, and again 24 h later. CFSE-labeled cells from draining LN were cultured with pBSDLs or with C-ter-J28 from different origins. After 6 days, CFSE dilution was analyzed by flow cytometry and T-cell proliferation evaluated. (C) Full-length pBSDL-OG1 immunization of mice led to a higher percentage of T-cells producing IFN-γ and granzyme B in culture with the immunizing antigen or its C-ter-J28. After 3 days, LN cells were labeled with anti-CD4 and -CD8 and analyzed for intracellular stainings of granzyme B and IFN-γ. (D) Culture supernatants were collected after 5 days for IFN-γ detection. The IFN-γ production by splenocytes cultured with pBSDL OG1 was greater than that with C-ter OG1, which correlates with the respective % of $CD4^+$ and $CD8^+$ T-cells expressing IFN-γ. The results are representative of two independent experiments.

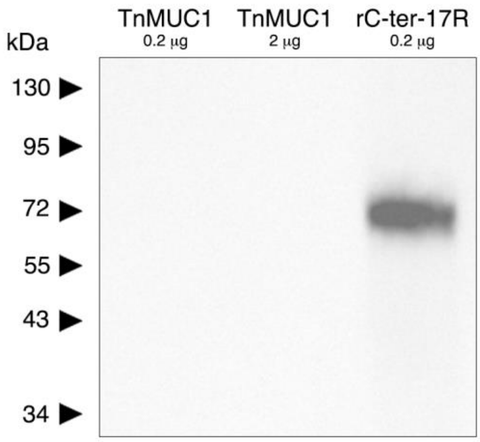

Supplementary figure S2: Detection of J28 expression in rC-ter-17R and in TnMUC1 by Western blotting. Glycopolypeptides (0.2 µg per lane unless indicated) were loaded on SDS-PAGE, transferred to nitrocellulose, and probed with mAbJ28. Representative immunoblot of three independent experiments.

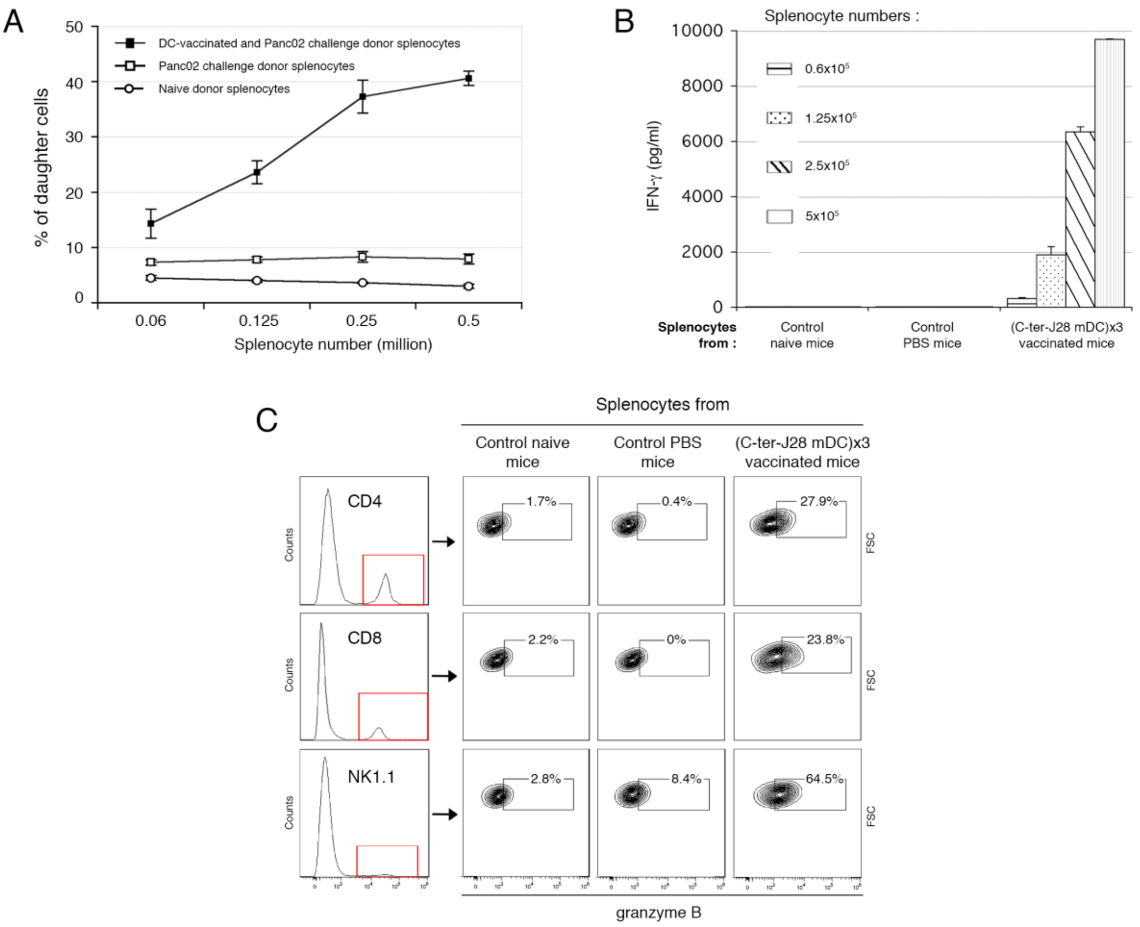

Supplementary figure S3: Immune status of mice protected from Panc02 tumor challenge. C57BL/6 mice were challenged SC with Panc02 4 days following the 3rd DC-injection. Splenocytes were collected 49 days after tumor challenge and plated at increased cellular density. (A) Proliferation of splenocytes from vaccinated and challenged mice, unvaccinated challenged mice and PBS-treated control mice. CFSE-labeled cells were cultured 4 days. CFSE dilution was analyzed by flow cytometry. (B) IFN-γ secretion**.** Culture supernatants were collected after 2 days of culture for IFN-γ detection. (C) Intracellular expression of granzyme B in $CD4^+$, $CD8^+$ and $NK1.1^+$ splenocytes from control and DC-vaccinated mice. Splenocytes were collected 36 days after tumor challenge and frozen. After thawing, living cells were plated at $2x10^5$ cells/well for five days.

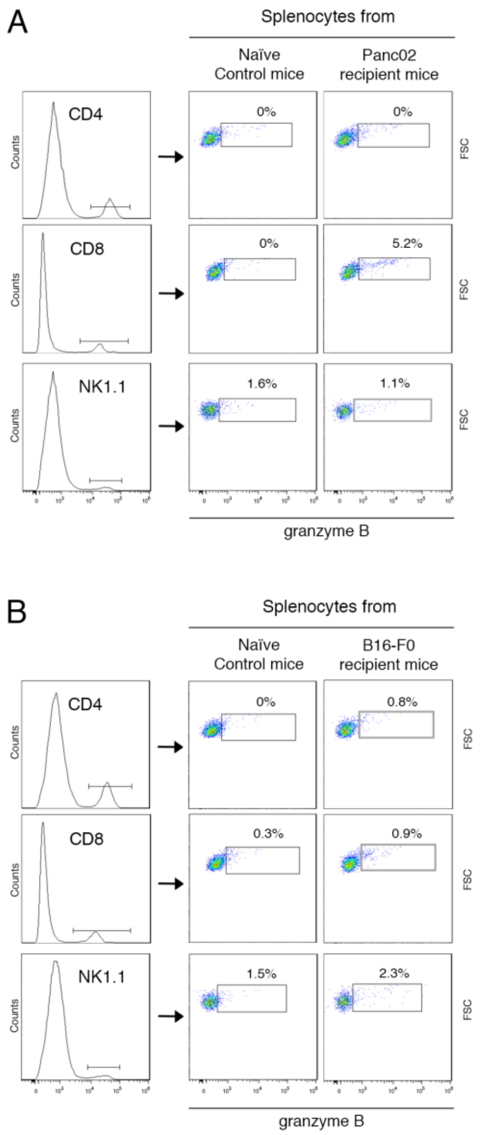

Supplementary figure S4: Immune status of mice inoculated with tumor cells. C57BL/6 mice were kept as naïve (A) or injected SC with 1x10^6 Panc02 cells (B) or 2.5x10^5 B16-F0 cells. Spleens from mice with well-developed tumors were collected. Intracellular expression of granzyme B in $CD4^+$, $CD8^+$ and NK1.1^+ cells was determined after 5 days of culture.

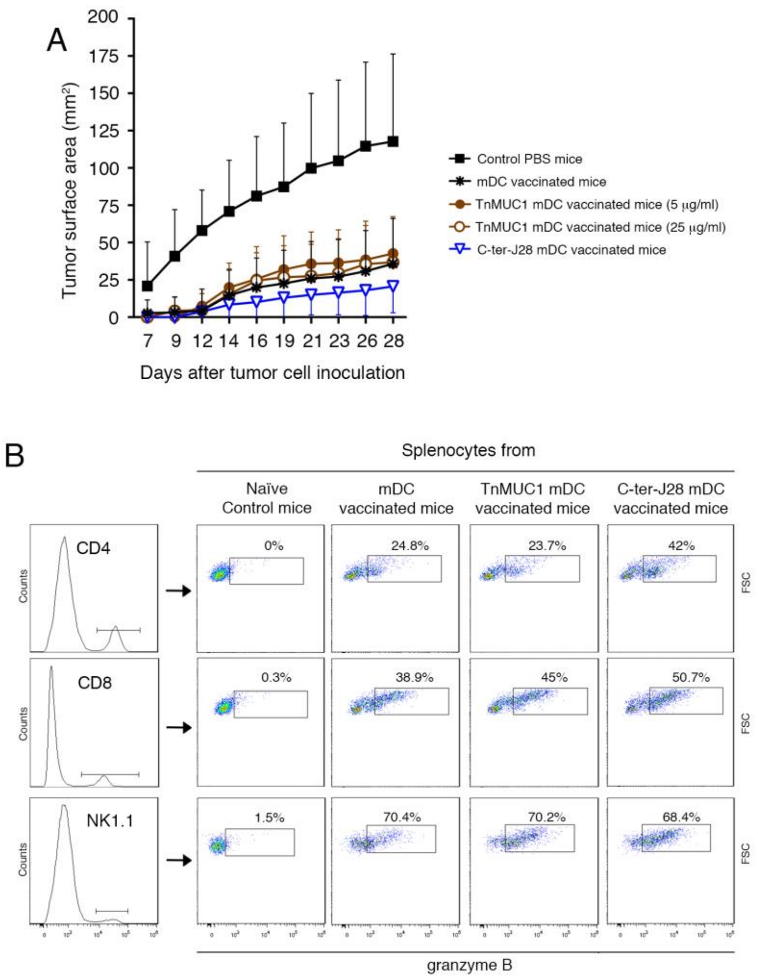

Supplementary figure S5: Effects of C-ter-J28 or TnMUC1 loading on DC in prophylactic vaccination. (A) C57BL/6J mice were either vaccinated SC with C-ter-J28^{+}-pulsed mDC, TnMUC1-pulsed DC or mDC three times at weekly intervals or they received PBS (control mice). Four days following the 3rd DC-injection, mice were challenged SC with Panc02 in the contralateral flank. Data are expressed as mean tumor surface area ± SEM. Comparisons between groups were made by two way-ANOVA repeated measurements, and differences were considered significant at $P<0.05$ (see Supplementary Table 3 for details). Control PBS mice, mDC vaccinated mice and C-ter-J28^{+} mDC mice: n=11; mice vaccinated with DC loaded with TnMUC1 (25 µg/ml; n=6); with TnMUC1 (5 µg/ml; n=5). (B) On day 28, spleens from protected DC-mice were collected. Intracellular expression of granzyme B in CD4^{+}, CD8^{+} and NK1.1^{+} cells was determined after 5 days of culture.

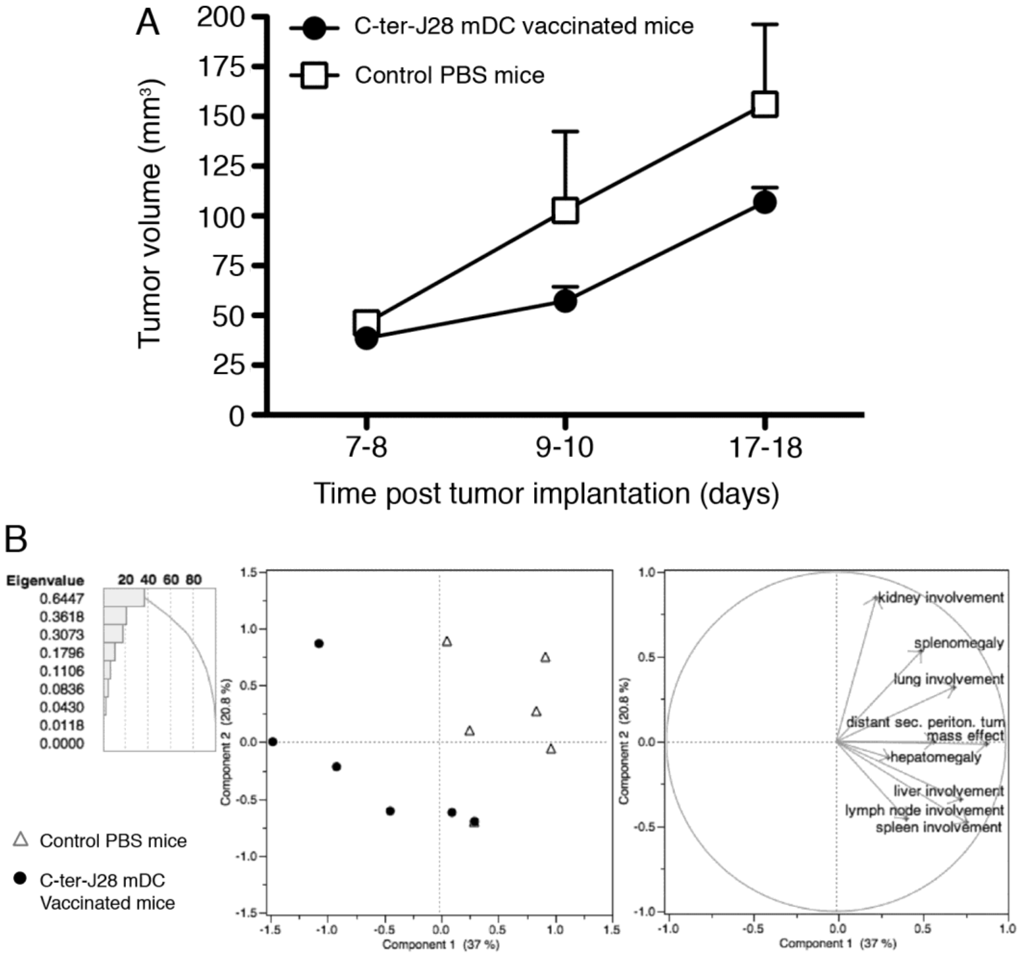

Supplementary figure S6: MRI analysis. Images were analyzed under ImageJ. Average tumor volumes (in mm^3) were computed by contouring the primary tumor mass containing the paper disc on all slices in all three planes and multiplying the obtained surface by 0.5/3. A qualitative disease progression scale with criteria based on the TNM staging system was used to account for local invasiveness, spread to abdominal LNs, distant peritoneal spread (to a different quadrant of the abdomen) and metastasis to other organs (spleen, kidneys, liver, lungs, subcutaneous tissue) as well as for the presence of ascites or a mass effect (Supporting Information Table S1). The disease progression scale used in this study varied from 0 to 14 (see Supporting Information Tables S1 and S2 for details on the scale). (A) *In vivo* assessment of primary tumor volume with contrast-enhanced MRI. A trend towards lower tumor volume was observed in the group of C-ter-J28-DC-vaccinated mice at days 9-10 and days 17-18 by

comparison to non-vaccinated mice, although it did not reach the statistical significance (vaccinated group: *n*=6 at day 7-8 and 9-10, *n*=3 at day 17-18; non vaccinated group: *n*=6 at day 7-8 and 9-10, *n*=3 at day17-18). (B) Principal components analysis (PCA) based on covariances of MRI findings used in the disease progression score. “Heterogeneity” and “local peritoneal tumors” were removed from the analysis since the vaccinated and non-vaccinated groups showed identical results. The 9 remaining correlated variables were transformed into 9 uncorrelated principal components obtained by eigenvalues decomposition of variable covariance matrix. The eigenvalues show the variance of each principal component. Principal component 1 and 2 show the greatest variance. The left graph gives the eigenvalue for each component as well as the corresponding percentage of variation (bar chart). The score plot allows visualization of the principal component points. This graph shows a separation between vaccinated mice (filled circle) and non-vaccinated mice (triangle). Principal components 1 and 2 explain 58% of the total variance. The loading plot on the right hand-side gives a 2D representation of factor loadings. Abbreviations: periton, peritoneal; sec, secondary; tum, tumor.

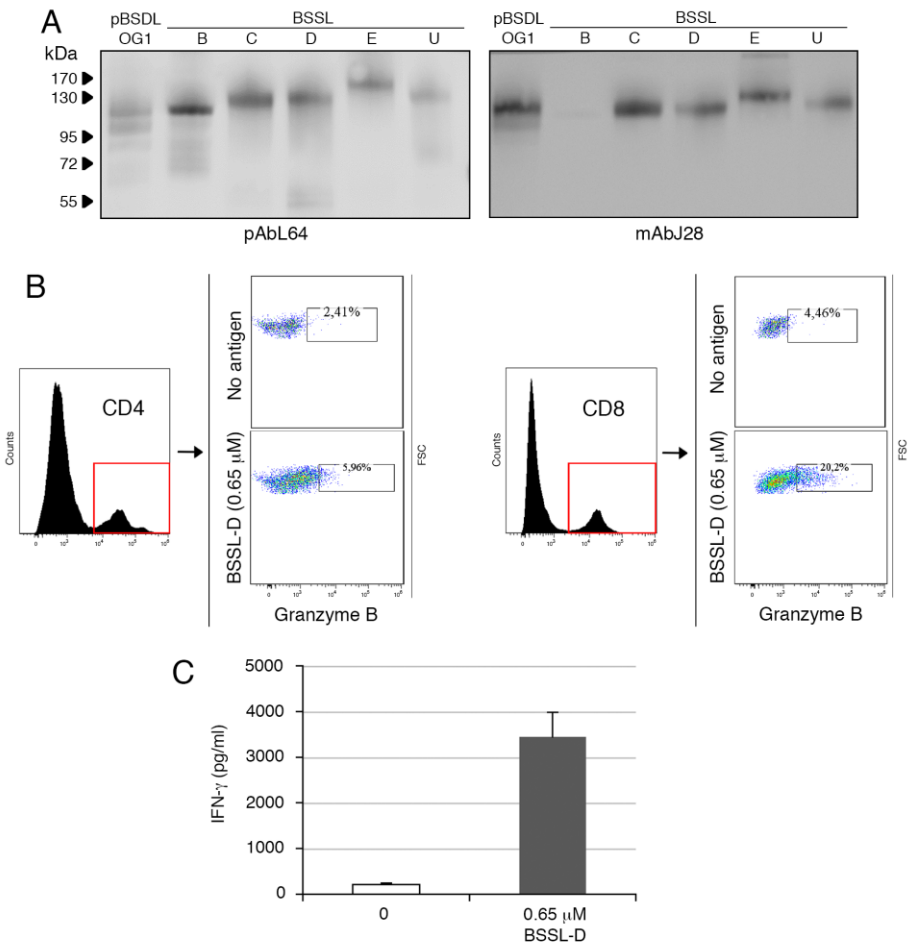

Supplementary figure S7: BSSLs as another source of J28+ glycoprotein. BSSLs -C, -D, -E and -U were purified from four human milk samples, and recombinant full length BSSL-B was purified from supernatants of human BSSL-transfected CHO cells. pBSDL-OG1 was used as positive control. (A) Immunodetection of BSSLs with pAbL64 and mAbJ28. Membranes were incubated with pAbL64 and mAbJ28 and then by peroxidase-labeled secondary Ab (Invitrogen, Cergy Pontoise, France) and revealed using appropriate substrate kits (Millipore Corp, Billerica, MA ; Promega Corp, Madison, WI). Native BSSLs showed similar immunoreactivity to pAbL64 and mAbJ28 as positive control pBSDL-OG1. Recombinant BSSL-B produced in CHO cells was reactive to pAbL64 but not to mAbJ28, concordantly with them not expressing glycosyltransferases involved in J28 epitope structure (13). (B-C) C57BL/6 mice were immunized with 50µg of pBSDL-OG1 and CFA. CFSE-labeled cells from draining LN were cultured with BSSL-D (0.65µM). Intracellular IFN-γ and

granzyme B staining was performed after 3 days of culture. Cell proliferation and percentage of intracellular stained cells were evaluated by FACS analysis. On the same day, culture supernatants were collected for IFN-γ detection. BSSL ability to *in vitro* activate T-cells from pBSDL-immunized mice is shown by increased percentages of granzyme B^+ $CD8^+$ T-cells (20.2% versus 4.46% without BSSL) (B) and by marked IFN-γ secretion (C).

Supplementary Table S1: Statistical analysis of the effects of C-ter-J28 or TnMUC1 loading on DC in prophylactic vaccination (see Supplementary figure S5)

	Significance (two-way ANOVA)						
	Days after tumor cell inoculation						
	7	9	12	14	16	19	21-28
Control PBS mice *vs* mDC vaccinated mice	ns	*	****	****	****	****	****
Control PBS mice *vs* TnMUC1 mDC vaccinated mice (5 μg/ml)	ns	ns	**	**	**	***	****
Control PBS mice *vs* TnMUC1 mDC vaccinated mice (25 μg/ml)	ns	*	**	**	***	***	****
Control PBS mice *vs* C-ter-J28 mDC vaccinated mice	ns	**	****	****	****	****	****

The statistical analysis was performed using the two-way analysis of variance (ANOVA) test followed by Bonferroni's multiple comparisons test.

* P <0.05; ** P <0.01; *** P <0.001; **** P <0.0001.

Supplementary Table S2 : Individual disease progression score

			primary tumor			locally advanced disease	involvement/metastasis						abdominal complications				
subject	day [a]	treatment	hetero-geneity	ill-defined margins	un-measurable	local secondary	lymph node	liver	spleen	kidney	lung	distant secondary	hepato-megaly	spleno-megaly	mass effect	ascites	total score
1	7 - 8	vaccinated	1	0	0	1	0	0	0	0	0	0	0	0	0	0	2
2			1	0	0	1	0	0	0	0	0	0	0	0	0	0	2
3			1	0	0	1	0	0	0	0	0	0	1	0	0	1	4
4			0	0	0	1	0	0	0	0	0	0	0	0	0	0	1
5			1	0	0	1	0	0	0	0	0	0	0	0	0	0	2
6			0	0	0	1	0	0	0	0	0	0	0	0	0	0	1
1	7 - 8	non vaccinated	1	0	0	1	0	0	0	0	0	1	0	0	0	0	3
2			1	0	0	1	0	0	0	0	0	0	0	0	0	1	3
3			1	0	0	1	0	0	0	0	0	1	0	0	0	0	3
4			1	1	0	1	1	0	1	1	0	1	0	0	1	1	9
5			0	1	0	1	0	0	0	0	0	0	0	0	0	0	2
6			1	0	0	1	0	0	0	0	0	0	0	0	0	1	3
1	9 - 10	vaccinated	1	0	0	1	1	0	0	0	0	0	0	0	0	0	3
2			1	0	0	1	0	0	0	0	0	0	0	0	0	0	2
3			1	1	0	1	0	0	0	0	0	0	1	0	0	1	5
4			1	1	0	1	0	0	0	0	0	0	0	0	0	1	4
5			1	0	0	1	0	0	0	0	0	0	0	0	0	1	3
6			1	0	0	1	1	0	0	1	0	1	0	0	0	0	5
1	9 - 10	non vaccinated	1	0	0	1	0	0	0	0	0	1	0	0	0	0	3
2			1	0	0	1	1	0	0	0	0	0	0	0	0	1	4
3			1	0	0	1	0	0	0	0	0	1	0	0	0	1	4
4			1	1	1	1	1	0	1	1	0	1	0	1	1	1	11
5			1	1	0	1	1	0	0	0	0	1	0	0	0	1	6
6			1	0	0	1	0	0	0	0	0	0	0	0	0	1	3
1	17 - 18	vaccinated	1	0	0	1	1	0	0	0	0	0	0	0	0	0	3
2			1	1	1	1	1	1	0	0	0	1	0	0	0	1	8
3			1	1	1	1	1	1	1	0	0	1	1	0	1	1	11
4			1	1	1	1	1	1	1	0	0	1	0	0	1	1	10
5			1	0	0	1	0	0	0	1	0	1	1	0	0	1	6
6			1	1	0	1	1	1	1	0	0	1	0	0	0	0	7
1	17 - 18	non vaccinated	1	1	1	1	1	1	1	1	1	1	1	0	1	0	12
2			1	1	1	1	1	1	0	1	0	1	0	1	1	1	11
3			1	0	0	1	1	1	1	0	1	1	1	1	1	1	11
4			1	1	1	1	1	1	1	1	1	1	0	1	1	1	13
5			1	1	0	1	1	1	1	1	0	1	0	0	1	1	10
6			1	0	0	1	1	1	1	0	0	1	1	0	1	1	9

[a] day after tumor transplantation

Supplementary Table S3: Correspondence of parameters used in the MRI-derived murine disease progression score with imaging-based clinical staging terminology

	murine disease progression score	imaging and clinical signs
primary tumor:	heterogeneity	mixed solid/cystic, partially enhancing with Gd-DOTA
	ill-defined margins	infiltrative growth pattern
	unmeasurable	unresectable due to numerous peritoneal extensions, multi-lobulated appearance
locally advanced disease:	local secondary	involvement of regional lymph nodes, peripancreatic tissues such as retroperitoneal and mesenteric fat and visceral peritoneum of adjacent organs = T4
metastasis:	lymph node	metastasis to non-regional lymph nodes = M1
	liver, spleen, kidney, lung	metastasis to distant organs = M1
	distant secondary	peritoneal seeding, distant retroperitoneal, gastric and subcutaneous metastasis = M1
abdominal complications:	hepatomegaly	hepatomegaly
	splenomegaly	splenomegaly, portal vein obstruction
	mass effect	organ displacement, bile duct obstruction, gall bladder distention
	ascites	ascites due to portal hypertension and/or metastasis

Abbreviations: M=metastasis, N=node, T=tumor.
